# Vector-borne zoonotic blood parasites in wildlife from Ecuador: A report and systematic review

**DOI:** 10.14202/vetworld.2021.1935-1945

**Published:** 2021-07-27

**Authors:** Eduardo Diaz, Anahi Hidalgo, Carla Villamarin, Gustavo Donoso, Veronica Barragan

**Affiliations:** 1 Escuela de Veterinaria, Hospital de Fauna Silvestre TUERI, Universidad San Francisco de Quito, Quito, Ecuador; 2 Colegio de Ciencias Biologicas y Ambientales, Instituto de Microbiologia, Universidad San Francisco de Quito, Quito, Ecuador; 3 Department of Biological Science, Pathogen and Microbiome Institute, Northern Arizona University, Flagstaff, USA

**Keywords:** amazon basin, blood parasites, Ecuador, free-ranging wildlife, hemoparasites, one health, systematic review, vector-borne zoonotic

## Abstract

**Background and Aim:**

Ecuador is a hugely diverse country, but information on infectious diseases in local wild animals is scarce. The aim of this study was to screen the presence of blood parasites in free-ranging wild animals admitted to the Wildlife Hospital at Universidad San Francisco de Quito, from April 2012 to January 2019.

**Materials and Methods:**

We identified blood parasites by microscopic observation of blood smears from free-ranging wildlife species that attended the Wildlife Hospital of Universidad San Francisco de Quito (Ecuador) from April 2012 to January 2019.

**Results:**

The microscopic evaluations of animals as potential reservoirs for vector-borne zoonotic blood parasites revealed the presence of *Anaplasma* spp., *Babesia* spp., *Ehrlichia* spp., *Hepatozoon* spp., microfilaria, *Mycoplasma* spp., and *Trypanosoma* spp. in previously unreported wildlife species. In addition, we performed a systematic review to understand the current knowledge gaps in the context of these findings.

**Conclusion:**

Our data contribute to the knowledge of blood parasites in wildlife from Ecuador. Furthermore, the potential transmission of these parasites to humans and domestic animals, current anthropogenic environmental changes in the region, and the lack of information on this suggest the importance of our results and warrant further investigations on infectious diseases in animals and humans and their relationship with environmental health as key domains of the One Health concept.

## Introduction

From an ecological perspective, a parasite lives in association with another organism [1]. Parasites that live in the host’s blood are called blood parasites (hemoparasites), which include several species of the genera *Anaplasma*, *Babesia*, *Ehrlichia*, *Haemoproteus*, *Hepatozoon*, *Leishmania*, *Leucocytozoon*, *Mycoplasma*, *Plasmodium*, *Schellackia*, and *Trypanosoma*, among others [2-6]. Usually, hosts become infected through arthropod vectors such as fleas, flies, mosquitoes, or ticks [7]. Blood parasites are globally distributed and infect a wide variety of amphibians [8,9], reptiles [10], birds [11,12], and mammals, including humans [13].

Parasitism is considered by some scientists as a malevolent condition where parasites survive at some cost to host health status [14]. Some parasites have coevolved with their hosts, permitting prolonged interactions, and minimum parasite damage [15]. However, when these parasites are transmitted from their original hosts to humans (zoonotic transmission), the infection may produce damaging pathology, potentially leading to severe diseases and even death [16]. Thus, wildlife species are natural reservoirs of a wide range of zoonotic parasites and may serve as sentinel species for emerging diseases [8,17]. In this context, the One health paradigm gives the cornerstones of the relationship between wildlife and human health. This concept encompasses the provision of health for humans, animals, and environment through the interactions between all three pillars. To date, the One health triad application is being emphasized to control, prevent, and monitor infectious diseases[18]. Currently, increasing research efforts have been concentrated on selected blood parasites, mainly *Leishmani*a spp., *Plasmodium* spp., and *Trypanosoma* spp. [7,18-21]. However, many vector-borne zoonotic hemoparasites from wildlife sources have been neglected; thus, information relating to their epidemiology and spillover related to human activity remains relatively uncharacterized [7,16-21].

In this study, we screened for blood parasites in blood smears from Ecuadorian free-ranging wildlife species admitted to the Wildlife Hospital at Universidad San Francisco de Quito, from April 2012 to January 2019. Furthermore, we complemented our findings with a systematic review of the literature investigating these parasites in wild animal species and their locations. The possible danger that the identified pathogens have in line with the One Health concept is also discussed, as some parasitic diseases are interconnected not only with animal health but also with human health through environmental relations [22].

## Materials and Methods

### Ethical approval

This study was conducted under the permit issued by Ecuador’s Ministry of Environment (019-2018-IC-FAU-DNB/MAE) and authorized by the Animal Ethics Committee of Universidad San Francisco de Quito USFQ (2018-011).

### Study period and location

Laboratory results reported in this study were compiled in February 2019 from animals admitted from April 2012 to January 2019. A database search for the systematic review was performed in June 2020. The study was performed in the Wildlife Hospital of Universidad San Francisco de Quito (Quito, Ecuador).

### Specimen selection/collection/identification

The laboratory results of blood samples belonged to free-ranging wildlife species from Ecuador were compiled. All animals were admitted from April 2012 to January 2019 to the Wildlife Hospital of Universidad San Francisco de Quito and sample collection was performed exclusively for infectious disease diagnostics. Samples were analyzed by Lab-Vet (permit Ref. No.: AGR-AGROCALIDAD/CDL-2018-000052-OF), using published and standard laboratory methods based on blood smear visualization by light microscopy using a 40X magnifying lenses.

### Literature review

PubMed (https://www.ncbi.nlm.nih.gov/pubmed), Scopus (https://www.scopus.com), and Web of Science (https://www.webofknowledge.com) databases were accessed and searched for published reports that match to our findings, search was performed on June 16, 2020. Search terms allowed us to retrieve original studies reporting *Anaplasma*, *Babesia*, *Ehrlichia*, *Hepatozoon*, microfilaria, *Mycoplasma*, and *Trypanosoma* in wild animal species. Search terms were used in all three databases to match the following combination of terms: (1) *Anaplasma*: Anaplasma AND ((Lagothrix AND lagotricha) OR (Saguinus AND fuscicollis) OR (Cebus AND albifrons)); (2) *Babesia*: Babesia AND ((Mazama AND rufina) OR (Lagothrix AND lagotricha) OR (Saguinus AND fuscicollis)); (3) *Ehrilichia*: Ehrilichia AND (Mazama AND rufina); (4) *Hepatozoon*: Hepatozoon AND (Melanosuchus niger OR Caiman crocodilus OR Boa constrictor); (5) *Microfilaria*: Microfilaria AND (M. niger OR Lagothrix lagotricha OR Nasua nasua); (6) *Mycoplasma*: ((Mycoplasma OR Haemobartonella) AND (N. nasua OR Panthera onca OR Puma concolor OR Leopardus tigrinus OR Leopardus pardalis OR Puma yagouaroundi OR Herpailurus yagouaroundi OR Potos flavus OR Lagothrix lagotricha OR Alouatta palliata OR Alouatta seniculus OR Cebus albifrons OR Sapajus apella OR Saimiri sciureus OR Saguinus fuscicollis OR Cebuella pygmaea OR C ebuella pygmaea OR Odocoileus virginianus OR Pudu mephistophiles OR Mazama rufina OR Choloepus didactylus OR Choloepus hoffmanni)); and (7) *Trypanosoma*: Trypanosoma AND (Choloepus AND hoffmanni). Searches were performed in July 2020 without publication year restrictions. Records in English, Portuguese, and Spanish were included in the study. The following were excluded from the study: (i) Studies that did not report the gender of blood parasites in animal species similar to this study, (ii) studies reporting vector-borne infections in animals other than the ones that we are reporting in this study (Table-1), (iii) studies conducted in laboratory animals or non-natural infections, (iv) duplicated titles or articles reporting results of other studies (ei. Reviews, not original research), and (v) studies that search for the parasites but do not find evidence of infection (negative reports) (Supplementary data can be available from the corresponding author). The country, publication year, blood parasite species, infected animal species, and diagnostic techniques (Supplementary data can be available from the corresponding author) were recorded for each manuscript.

**Table-1:** Hemoparasites found in blood smears of free-ranging wildlife animals attended at HDEV-USFQ.

Order	Family	Animal species	Anaplasma	Babesia	Ehrlichia	Hepatozoon	Microfilarias	Mycoplasma	Trypanosoma
Carnivora	Felidae	*Puma yaguarondi*						1	
		*Leopardus pardalis*						15	
		*Leopardus tigrinus*						2	
		*Puma concolor*						6	
		*Panthera onca*						3	
	Procyonidae	*Nasua nasua*					1	2	
		*Potos flavus*						2*	
Primates	Atelidae	*Lagothrix lagotrichia*	1*A	1*			1*	5*A	
		*Alouatta palliata*						1*	
		*Alouatta seniculus*						1*	
	Cebidae	*Cebus albifrons*	2*B					13*B	
		*Sapajus apella*						1	
		*Saimiri sciureus*						12	
	Callitrichidae	*Saguinus fuscicollis*	1*	1*				10*	
		*Callithrix pygmaea*						1*	
Artiodactyla	Cervidae	*Odocoileus virginianus*						1*	
		*Pudu mephistophiles*						1*	
		*Mazama rufina*		1*C	2*C			1*C	
Pilosa	Megalonychidae	*Choloepus didactylus*						1*	
		*Choloepus hoffmanni*						1*	
Crocodilia	Alligatoridae	*Melanosuchus niger*				5D	3*D		
		*Caiman crocodilus*				3			
Squamata	Boidae	*Boa constrictor*				2			

* First time reported

A Mycoplasma sp. and Anaplasma sp.found in the blood smear of the same animal.

B Both, Mycoplasma sp. and Anaplasma sp. was found in the blood smear of two animals.

C Mycoplasma sp., Babesia sp. and Ehrlichia sp found in blood smear of the same animal.

D Microfilaria and Hepatosoon was found in the blood smear of three animals.

## Results

### Hemoparasites in blood samples from free-ranging wildlife species

From April 2012 to January 2019, 290 free-ranging wildlife animals attended our veterinary facility, including 209 mammals, 52 reptiles, and 29 birds (Supplementary data can be available from the corresponding author). Blood parasites were detected in 97 (33.45%) animals, of which 87 were mammals and ten were reptiles. No positivity was identified in birds.

*Anaplasma* spp. was registered in four samples from three nonhuman primate species: Two White-fronted capuchin (*C. albifrons*) and one Brown woolly monkey (*L. lagotricha*) demonstrated coinfection with *Mycoplasma* spp. and also one Brown-mantled tamarin (*S. fuscicollis*) (Table-1). *Babesia* spp. was found in three mammal species: One Brown woolly monkey, one Brown-mantled tamarin, and one Little red brocket (*M. rufina*) that showed coinfection with *Mycoplasma* spp. and *Ehrlichia* spp., respectively. We also identified another Little red brocket infected with *Ehrlichia* spp. *Mycoplasma* spp. was the most frequent hemoparasite and was identified in 80 (91.95%) of the positive samples from 20 different mammal species (Table-1). *Mycoplasma* was followed by *Hepatozoon* spp. and was found in samples from ten reptiles (five Black caiman - *M. niger*, two snakes *B. constrictor*, and three Spectacled caimans - *C. crocodilus*). Three Black caiman showed coinfection with microfilariae. Microfilaria was also found in a South American coati (*N. nasua*) and a Brown woolly monkey. Finally, we identified one Hoffmann’s two-toed sloth (*C. hoffmanni*) positive for *Trypanosoma* spp. (Table-1).

### Literature searches to investigate hemoparasites in animal species reported in this study

In total, 41 reports matched the hemoparasite findings in the 23 free-ranging wildlife species reported here (Supplementary data can be available from the corresponding author). Twelve studies reported more than one parasite or infected wild animal [23-34]. The search yielded studies from1979 to 2019, with Brazil having the most studies (n=26). The remaining studies were conducted in the USA (n=4), French Guiana (n=4), Colombia (n=2), Mexico (n=2), Costa Rica (n=2), and Peru (n=1).

Our literature review identified 16 *Hepatozoon* studie*s* that matched our reptile findings. Reports of *Hepatozoon fusifex*, *Hepatozoon terzii*, and *Hepatozoon cuestensis* were identified for *B. constrictor* and *Hepatozoon caimani* for *C. crocodilus* and *M. niger*. The most common technique used to detect these parasites was the microscopic observation of blood smears. Electron microscopy and molecular detection (followed by sequencing) techniques were also used to identify *Hepatozoon* species [34-36]. For *Mycoplasma*, various species were previously reported in nine out of the21 searched mammal species (Table-2) [3,23,25-34,37-56]. *Mycoplasma* spp. detection and identification were primarily conducted using polymerase chain reaction (PCR) and sequencing. For *Trypanosoma* species, *Trypanosoma* *leeuwenhoek* and *Trypanosoma* *rangeli* were isolated and identified in *C. hoffmanni* using blood smear microscopic observations, histopathology, parasite culture, and PCR. Finally, microfilariae were previously observed in *N. nasua* and *M. niger* blood smears. Microfilaria in samples from *N. nasua* was identified as *Dirofilaria repens*, *Dirofilaria immitis*, *Acanthocheilonema reconditum*, *Mansonella* spp., and *Brugia* spp.

**Table-2:** Hemoparasites that have been previously reported in free-ranging wildlife animals.

Hemoparasite	Animal
*Hepatozoon* spp. [3,34,37-44]	*Caiman crocodilus, Boa constrictor*
*Hepatozoon caimani* [45,46]	*Caiman crocodilus*
*Hepatozoon fusifex* [47]	*Boa constrictor*
*Hepatozoon terzii* [48]	*Boa constrictor*
*Hepatozoon cuestensi* [49]	*Boa constrictor*
*Mycoplasma* spp. [26, 50]	*Puma concolor, Odocoileus virginianus*
*Mycoplasma haemocanis* [28]	*Nasua nasua*
*Mycoplasma haemofelis* [23,25, 32-33,51]	*Nasua nasua, Pantera onca, Puma concolor, Leopardus tigrinus*
*Mycoplasma haemocanis/Mycoplasma haemofelis* [28,32]	*Leopardus pardalis*
*Candidatus Mycoplasma turicensis* [23,32,51]	*Nasua nasua, Pantera onca, Leopardus pardalis*
*Candidatus Mycoplasma haemominutum* [23,25,26,29-32]	*Pantera onca, Puma concolor, Leopardus tigrinus, Leopardus pardalis*
*Candidatus Mycoplasma haemomacaque* [27]	*Sapajus apella*
*Candidatus Mycoplasma kahaneii* [27,30]	*Saimiri sciureus*
*Trypanosoma leeuwenhoek* [52]	*Choloepus hoffmanni*
*Trypanosoma rangeli* [53,54]	*Choloepus hoffmanni*
*Dirofilaria repens* [55]	*Nasua nasua*
*Dirofilaria immitis*[55]	*Nasua nasua*
*Acanthocheilonema reconditum* [55]	*Nasua nasua*
*Mansonella* spp. [55]	*Nasua nasua*
*Brugia* spp. [55]	*Nasua nasua*
Non identified mirofilaria species [56]	*Melanosuchus niger*

Our literature search did not identify *Hepatozoon* spp. in *M. niger* or *Mycoplasma* spp. in *P. flavus*, *L. lagotricha*, *A. palliata*, *A. seniculus*, *C. albifrons*, *P. mephistophiles*, *S. fuscicollis*, *C. pygmaea*, *M. rufina*, *C. didactylus*, or *C. hoffmanni*. In addition, no publications reported the presence of *Babesia* spp. in *M. rufina*, *L. lagotricha*, or *S. fuscicollis* or *Ehrlichia* in *M. rufina*.

## Discussion

The detection of vector-borne blood parasitized wildlife species may provide valuable information on the transmission cycles of pathogens in nature. Knowing the potential parasite reservoirs could inform authorities to implement preventative measures to reduce the risk to wildlife, domestic animal, and human health [57]. From a public health perspective, this approach should be of particular relevance to environments with high animal diversity, where habitat fragmentation has affected the abundance and distribution of vectors and reservoirs involved in the transmission cycle of zoonotic parasites [58].

This study reported vector-borne zoonotic blood parasites in several wild mammal and reptile species from Ecuador. The findings were complemented by data from a systematic review where we searched studies of hemoparasites that infect the wild species identified in our work. For the first time, we report the occurrence of *Anaplasma*, *Babesia*, *Ehrlichia*, *Hepatozoon*, and *Mycoplasma* in various wild animal species. These data suggest knowledge gaps of infectious diseases circulating in wildlife from different ecosystems in our region.

All hemoparasites identified here could infect numerous animal species, including humans, and may be biologically or mechanically transmitted by arthropods. Anaplasmosis and ehrlichioses are two tick-borne infectious diseases and are well known in veterinary medicine circles and were recently shown to cause mild, moderate, and severe disease in humans [59]. Both bacteria are obligate intracellular pathogens vectored by ticks, which serve as vectors for vertebrate hosts [60,61]. Babesiosis is an emerging zoonotic disease caused by tick-borne apicomplexan protozoa of the genus *Babesia* [62]. This pathogen infects a wide range of animals, with wildlife species being the principal reservoir hosts for zoonotic *Babesia* species [63]. *Hepatozoon* spp. is intraerythrocytic tick-borne protozoa; infections occur when an infected invertebrate host is ingested by a vertebrate host. Infection with Hepatozoon spp. can affect birds mammals and reptiles and can produce mild to severe disease including symptoms such as anorexia, weight loss, fever, and polyuria, or remain asymptomatic [64-66]. Humans infected with *Hepatozoon* have been reported [67], suggesting a possible but rare infection incidence. Filarial nematodes are vector-borne worms that release microfilariae into the host blood; these pathogens are considered to be a threat for animal and human health [68]. Most have adapted to the transmission through hematophagous arthropods [69] and can be found in surgical tissue biopsy specimens or removed intact from superficial sites from infected animals [70]. Globally, *Mycoplasma* spp. represent emerging pathogens in animals and humans, with wildlife animals being important bacterial reservoirs [71,72]. These organisms are epicellular erythrocytic pathogens, transmitted by blood-feeding arthropod vectors [73,74] and causing asymptomatic to mild and severe disease in a wide variety of mammalian species [75]. Finally, trypanosomes, the most common hemoparasites in the world, are transmitted by hematophagous invertebrate vectors [76]. They infect humans and domestic and wild animal species, often causing chronic and fatal diseases [77].

The most common hemoparasite observed in this study was *Mycoplasma*, infecting a variety of wildlife species from Artiodactyla, Carnivora, Crocodilia, Pilosa, Primates, and Squamata orders (Table-1). Multiple *Mycoplasma* species have been reported in *Nasua* spp., including coinfection with *Trypanosoma cruzi* in *Nasua narica* from Costa Rica [78]. We also observed this microorganism in *Odocoileus virginuanus*. About Mycoplasma, the two species that commonly infect cervids are *M. ovis* and *M. haemocervae*, and we were able to identified a report that describes a genetically closely related species to *M ovis* infecting *O. virginuanus* [79]. In addition, *Mycoplasma* infection in free-ranging felids was higher than in wild felids from zoos [26]. Several studies described *Mycoplasma* circulation among domestic cats and free-ranging felids [26,80,81]. Here, we reported for the first time the presence of *Mycoplasma* in 27 wild felids from Ecuador. These felids belonged to five different species, which are all free-ranging animals. Felids are important top predators; thus, the identification of hemoparasites infections may suggest these animals are bioaccumulating pathogens from prey species [82]. This may be why we did not find *Mycoplasma* in *M. niger*. Likewise, *Mycoplasma* reports were not identified for *A. palliata* and *A. seniculus*. However, this was expected as previous studies have reported the presence of *Candidatus Mycoplasma kahan*e in *Alouatta caray*a and *Sapajus flavius* [24,74]. Our systematic review found that *Mycoplasma* was not reported in species such as *Choloepus* spp*., Lagothrix lagotricha*, *Mazama rufina*, *P. flavus*. and *P. mephistophiles* among others, agreed with our laboratory findings. This information identified knowledge gaps in the prevalence and dynamics of *Mycoplasma* species in wildlife animals.

Despite *Trypanosoma* spp. being one of the most common hemoparasite genera in wildlife [83-86] and its repeated identification in multiple sloth species [3,54,87-89], we identified it only in *C. hoffmanni*. For microfilaria species, they were previously reported in *M. niger* and *N. nasua*. In the latter, reports of *D. repens*, *D. immitis*, *A. reconditum*, *Mansonella* spp., and *Brugia* spp. suggested its importance as a reservoir, while the filaria infecting *M. niger* remains known [55]. Here, we reported for the first time the presence of microfilaria in *L. lagotricha*. However, further studies are required to identify filaria diversity infecting other wildlife species, including *M. niger* and *L. lagotricha*.

Hepatozoon mainly infects reptiles and, to a lesser extent, mammals [3,90]. We observed this genus in the reptiles, *C. crocodilus*, *B. constrictor*, and *M. niger*. Interestingly, only *H. caimani* was identified in *Caiman* spp. This result is notoriously contrasting with the reports of a high diversity of Hepatozoon species *(H. fusife*x, *H. terzi*i, and *H. cuestensi*s) that could infect *B. constricto*r [43,91-93]. We also reported the presence of *Ehrlichia* in *M. rufina*, even though we were unable to identify previous reports of this infection. However, *Ehrlichia* studies exist for other deer species [94]. Likewise, we did not find reports of *Anaplasma* in *S. fuscicollis*, *C. albifrons*, and *L. lagotricha* nor reports of *Babesia* in *S. fuscicollis*, *L. lagotricha*, and *M. rufina*, although these two hemoparasites have been reported in other nonhuman primates and ruminants [95-97]. Therefore, these data contribute to the distribution knowledge of these hemoparasites.

The analytical methodology used in our study was based on blood smear visualization using light microscopy, a method traditionally used for blood parasite diagnostics in veterinary medicine [98]. Although microscopic diagnostic sensitivity and specificity for this method are generally lower than PCR [71], it generates fewer false positives, which is often a major concern in large-scale PCR studies [99,100]. However, the microscopic identification of some blood parasites, for example, *Babesia* spp. [101], *Hepatozoon* spp. [102], or *Microfilaria* spp. [68], remains the “gold standard” diagnostic tool. Therefore, optical microscopy could be a simple and low-cost method for determining hemoparasite prevalence in wildlife [103-105]. We readily acknowledge that the results from this research may be underestimated due to our favoring only microscopic techniques. Therefore, further studies using combined morphological and molecular analyses are required to provide more precise identifications of blood parasites in wildlife [95,103-104].

An interesting finding from this study was that most wildlife species (22 out of 23), where zoonotic blood parasites were detected, were distributed in the Amazon Basin: *Alouatta. seniculus* [105], *B. constrictor* [106], *C. crocodilus* [107], *C. pygmaea* [108], *C. albifrons* [109], *Cebus macrocephalus* [110], *C. didactylus* [111], *C. hoffmanni* [112], *L. lagotricha* [113], *Leopardus pardalis* [114], *L. tigrinus* [115], *M. rufina* [116], *M. niger* [117], *N. nasua* [118], *O. virginianus* [119], *P. onca* [120], *P. flavus* [121], *P. mephistophiles* [122], *P. concolor* [123], *P. yagouaroundi* [124], *S. fuscicollis* [125], and *S. sciureus* [126]. Only *A. palliata* is not distributed in the Amazon basin, it lives in the Choco Tropical Rain Forest, and in the deciduous and piemontan forest of the Western Cordillera of the Ecuadorian Andes [127].

The Amazon Basin comprises many of the most diverse global ecosystems across seven countries, namely, Bolivia, Brazil, Colombia, Ecuador, Guyana, Peru, and Venezuela [128]. These complex ecosystems contain natural foci of vector-borne human parasitic diseases. However, disease dynamics have been greatly affected due to deforestation and climate change [129,130]. Vector-borne diseases are particularly sensitive to climate warming, as arthropod vector development rates, geographical distribution, and transmission dynamics are altered [131,132]. Along with increased global temperatures, anthropogenic factors, such as increased urbanization, agriculture, and livestock, are invading wildlife habitats, with increased interactions between humans, domestic, and wild animals[133,134]. These changes in wild ecosystems create new opportunities for vector establishment or increases in existing populations [7,20,101,135]. Specifically, Ecuador has suffered the highest deforestation rate in South America [136], with evidence showing that 46% of South Ecuador’s original forest cover had been converted by 2008 into pastures and other anthropogenic land covers [137]. Furthermore, projected models for future climate change scenarios in Ecuador have demonstrated potential shifts in mosquito distribution, that could lead to an increase of vector-borne infectious diseases [138].

Our systematic review revealed a dearth of publications on selected hemoparasites in the animal species we identified from the Amazon Basin. Specifically, no publications emanated from Bolivia, Ecuador, and Venezuela, whereas most of the research came from Brazil. Therefore, an important vector-borne zoonotic blood parasitology research gap exists in these regions. For these reasons, research efforts are required in these regions where current environmental changes could lead to new epidemiological patterns in vector-borne disease, generating negative impacts on wildlife, domestic animals, and human health. Indeed, pathogen transmission from a vertebrate animal to a human (zoonotic spillover) represents a global but poorly understood public health issue [134]. Thus, a better understanding of the evolution and ecology of zoonotic blood parasites is crucial to determine the foci of possible emerging infectious diseases and to prevent the outbreak of diseases [139,133]. Therefore, as other authors have indicated [38,129], epidemiological surveillance is required to address vector-borne zoonotic blood parasites in the Amazon Basin and other highly diverse ecosystems. In addition, as interfaces between wildlife, domestic animals, and human populations interact and become more complex, more interdisciplinary collaborations will be required to prevent disease transmission at these junctures [140,141]. The only way authorities and policymakers will make informed decisions and prioritize action issues is to foster projects framed under the one health initiative, contributing to public health improvement, wildlife conservation, and livestock sustainability through this holistic strategy [142].

## Conclusion

We described vector-borne zoonotic blood parasite diversity in wildlife species from Ecuador and highlighted the lack of information in the region. Indeed, zoonotic spillover is a poorly understood public health issue; thus, our work emphasizes how research can determine if infected wildlife are important reservoirs of hemoparasites distinct to the common *Leishmania* spp., *Plasmodium* spp., and *Trypanosoma* spp. In addition, the fact that all the blood parasites identified in this work are transmitted by arthropod vectors suggests that their ecology and distribution may be affected by habitat fragmentation and climate change. Our data add relevant information to the literature on infectious diseases in wildlife and spotlights the need to understand diseases and its consequences in animal and human health within ecosystem conservation and dynamics, which are the pillars of the one health framework.

## Authors’ Contributions

ED: Conceptualization, methodology, writing – original draft, review, and editing. AH, CV, GD: Methodology, data compilation. VAB: Conceptualization, methodology, writing – review and editing, and supervision. All authors have read and approved the final manuscript.
